# Species Delimitation, Phylogenetic Relationships, and Temporal Divergence Model in the Genus *Aeromonas*

**DOI:** 10.3389/fmicb.2018.00770

**Published:** 2018-04-20

**Authors:** J. G. Lorén, Maribel Farfán, M. C. Fusté

**Affiliations:** ^1^Departament de Biologia, Sanitat i Medi Ambient, Secció de Microbiologia, Facultat de Farmàcia i Ciències de l’Alimentació, Universitat de Barcelona, Barcelona, Spain; ^2^Institut de Recerca de la Biodiversitat, Universitat de Barcelona, Barcelona, Spain

**Keywords:** species delimitation, GMYC, diversification model, *Aeromonas*, *mdh*, *recA*

## Abstract

The definition of species boundaries constitutes an important challenge in biodiversity studies. In this work we applied the Generalized Mixed Yule Coalescent (GMYC) method, which determines a divergence threshold to delimit species in a phylogenetic tree. Based on the tree branching pattern, the analysis fixes the transition threshold between speciation and the coalescent process associated with the intra-species diversification. This approach has been widely used to delineate eukaryote species and establish their diversification process from sequence data. Nevertheless, there are few examples in which this analysis has been applied to a bacterial population. Although the GMYC method was originally designed to assume a constant (Yule) model of diversification at between-species level, it was later evaluated simulating other conditions. Our aim was therefore to determine the species delineation in *Aeromonas* using the GMYC method and asses which model best explains the speciation process in this bacterial genus. The application of the GMYC method allowed us to clearly delineate the *Aeromonas* species boundaries, even in the controversial groups, such as the *A. veronii* or *A. media* species complexes.

## Introduction

As in other organisms, diversification in bacteria leads to entities that we call species, which group together organisms that have evolved separately from others. Species can be differentiated from populations of individual strains by a maximum likelihood method that determines the point of transition of the evolutionary processes from the level of species (speciation and extinction) to the population (coalescence). The entities determined in this way maintain the biological properties and the levels of sequence divergence of the traditionally defined species.

Several methods have been proposed for species delimitation in bacteria. The classical approach uses a sequence divergence of 3 per cent pairwise distance in 16S rRNA (Schloss and Handelsman, 2006) or the 1 percent recommended by Acinas et al. (2004) as a threshold for separating species. However, rates of substitution may vary among lineages, as can the levels of variation within and between species, which makes it difficult to assume a universal threshold. Other widely applied methods for species delimitation are based on multilocus sequences, or more recently whole genome sequencing of several individuals has been used to determine the average nucleotide identity (ANI) (Richter and Roselló-Mora, 2009) and *in silico* DNA-DNA hybridization (*is*DDH) (Meier-Kolthoff et al., 2013) values to separate species. When comparing these methods with traditional phenotypic approaches, discrepancies can sometimes arise.

The study of evolutionary patterns in prokaryotes is hampered by the lack of a reliable fossil record, limited morphological differentiation and frequently complex taxonomic relationships. The few studies in this field suggest a constant rate of diversification in free-living or symbiotic bacteria (Martin et al., 2004; Vinuesa et al., 2005; Sanglas et al., 2017), while in some pathogens, such as *Borrelia burgdorferi*, it seems to follow a radiation pattern (Morlon et al., 2012). Differences in the cladogenesis of *B. burgdorferi* could be explained by its association with vertebrate and arthropod hosts, which could restrict the gene flow between populations. Understanding the evolution of prokaryote biological diversity therefore remains a significant challenge for biologists (Barraclough et al., 2009).

The main objective of our research in the last years has been the study of diversification in the genus *Aeromonas*, a γ-*Proteobacteria* that comprises a group of Gram-negative, rod-shaped bacteria, which are found in aquatic environments worldwide and are members of the microbiota (as well as primary or secondary pathogens) of fish, amphibians and other animals (Martin-Carnahan and Joseph, 2005; Janda and Abbott, 2010). *Aeromonas* is an ideal genus to study the diversification processes in bacteria because its species, a combination of free-living bacteria and host-associated strains, can be isolated from a wide variety of habitats. We began by investigating the rate and pattern of cladogenesis in this bacterial genus from a collection of strains including the type strains of all the *Aeromonas* species, using the sequences of five housekeeping genes (Lorén et al., 2014). However, the frequently high intra-specific diversity in bacteria is not always adequately reflected by the type strain.

We therefore performed a second analysis using molecular data from the sequences of two housekeeping genes (*mdh* and *recA*) obtained from 150 strains belonging to 27 species of *Aeromonas* (Sanglas et al., 2017). Phylogenies that mix variation between and within species often need to be reduced to trees with only one sequence per species to avoid invalidating the results (Fontaneto et al., 2012). To fulfill these conditions we constructed a tree with the consensus sequence for each species and used the BEAST program to obtain the species tree (Sanglas et al., 2017). In both cases the results of the analysis allowed us to determine that the process of speciation in *Aeromonas* follows a constant model of diversification.

The genus *Aeromonas*, includes 30 taxonomic species, many of them described recently. However, some descriptions were based on only one strain, or using techniques such as the 16S rRNA gene sequences, which have been demonstrated to have a low discriminatory power for delimiting species in *Aeromonas* (Figueras et al., 2000). Consequently, the improper characterization of some *Aeromonas* species has led to several reclassifications, as in the case of *A. aquariorum* (Beaz-Hidalgo et al., 2013), *A. ichthiosmia* (Huys et al., 2001), *A. culicicola* (Huys et al., 2005), among others. In addition, the genus includes several species complexes, such as *A. hydrophila* (Martin-Carnahan and Joseph, 2005), *A. veronii* (Silver et al., 2011) or *A. media* (Talagrand-Reboul et al., 2017), which are constituted by groups of strains with high intra-specific heterogeneity, giving rise to controversies about the species delineation.

In this study, we aimed to determine the species delineation and corroborate the diversification process in *Aeromonas* by applying a sequence-based method that allows the use of several strains per species, the Generalized Mixed Yule Coalescent (GMYC) method. This approach has been widely used to delineate eukaryote species and establish the diversification process from sequence data (Pons et al., 2006; Fontaneto et al., 2007; Lahaye et al., 2008). Nevertheless, there are only a few previous examples in which this analysis has been applied to a bacterial population (Barraclough et al., 2009; Powell, 2012). The method determines a divergence threshold to delimit species in a phylogenetic tree. Based on the tree branching pattern, the analysis fixes the transition threshold between speciation and the coalescent process associated with the intra-species diversification. The pruned tree provided by the threshold allows the use of classical methods of diversification analysis to determine the speciation process followed by the species in the analyzed population.

It is presupposed that branch lengths between species are derived from speciation and extinction rates, whereas branch lengths within a species reflect a coalescence process at the population level (Pons et al., 2006). The method determines the locations of ancestral nodes that define putative species and applies a likelihood ratio test to assess the fit of the branch lengths to a mixed lineage birth-population coalescent model (Pons et al., 2006). As the analysis to determine the transition threshold is based on the tree branching pattern, the tree has to accomplish certain requirements, for example, be ultrametric, fully resolved (without multifurcations) and reliable (with a high support in the nodes).

Our aim was therefore to determine the species delineation in *Aeromonas* based on the GMYC method. Although initially this method assumed diversification at between-species level follows a constant (Yule) model (Pons et al., 2006; Fontaneto et al., 2007), it was later evaluated simulating other diversification models (Fujisawa and Barraclough, 2013). Despite previous results indicate that *Aeromonas* follows a constant (Yule) model of diversification (Lorén et al., 2014; Sanglas et al., 2017), we also determined which model best explains the speciation process in this bacterial genus.

## Materials and Methods

### Gene Sequences

A collection of 147 *Aeromonas* strains, representative of the 30 species recognized up to August 2017, was selected for the study. Two housekeeping genes (*mdh* and *recA*) were chosen for the analysis; for each strain, the full-length sequences for both genes were previously obtained (Sanglas et al., 2017). In the case of the more recently accepted *Aeromonas* species: *A. lacus, A. aquatica* and *A. finlandiensis*, sequences were obtained from genomes available in the GenBank^1^. The strains and sequences are listed in Supplementary Table S1, including the GenBank accession numbers.

### Data Sets

Phylogenetic reconstruction was carried out from the concatenated sequences of *mdh* and *recA* genes. For each gene, the translated sequences were aligned using the ClustalW program implemented in MEGA6 (Tamura et al., 2013) and translated back to obtain the nucleotide alignments. Both alignments were concatenated with the DAMBE program (v5.3.10; Xia, 2013) and later the sequences were checked for the presence of incongruences or gaps. Divergent and ambiguously aligned blocks were also removed using the Gblocks program (Castresana, 2000).

### Sequence Analysis

To ascertain if the sequences used allowed a good species discrimination we determined the intra- and inter-specific distances among the different *Aeromonas* species. Data were graphically depicted with box plots obtained using the R package ggplot2 (Wickham, 2016). Sequences were also analyzed with different distance models that consider the influence of saturation, base frequency, transition–transversions or multiple substitutions. All the analyses were conducted with the function dist.dna implemented in the R package ape (Paradis et al., 2004). To determine the polymorphic sites along the sequences we construct a dots plot graph with the R package phyclust (Chen, 2010, 2011). The R package NbClust (Charrad et al., 2014) was used to determine the number of clusters from the *p* distances of the alienated sequences (Supplementary Table S2).

### Phylogenetic Reconstruction

Bayesian phylogenetic trees were reconstructed with the BEAST program (v1.8.1; Drummond and Rambaut, 2007; Drummond et al., 2012) from the data sets. The model of evolution for the each gene was determined using the jModelTest 2 program (Darriba et al., 2012). The general time-reversible model with discrete gamma distribution and invariant sites (GTR + G + I) was selected as the best-fit model of nucleotide substitution. The Bayesian analysis was performed using a GTR model with four gamma categories, a Yule process of speciation, and a constant clock model of evolution as the tree priors, as well as other default parameters. We used the divergence time between *Escherichia coli* and *Salmonella enterica* estimated by Ochman and Wilson as the calibration point (Ochman and Wilson, 1987a,b). Accordingly, we calibrated the divergence of *Aeromonas* with a normally distributed prior with a mean of 140 Ma and a standard deviation of 10 Ma. We performed three independent Markov Chain Monte Carlo (MCMC) runs 20 million generations, sampling every 2,000 generations. Posterior distributions for parameter estimates and likelihood scores to approximate convergence were visualized with the Tracer program (v1.6.0; Rambaut et al., 2014). Visual inspection of traces within and across runs, as well as the effective sample sizes (ESS) of each parameter (>200), allowed us to confirm that the analysis was adequately sampled. A maximum clade credibility (MCC) tree was chosen by TreeAnnotator (v1.8.1; Drummond et al., 2012) from the combined output of the three MCMC runs using the LogCombiner program^2^ after the removal of the initial trees (20–25%) as burn-in. The MCC tree was visualized with the program FigTree (v1.4.2)^3^.

### GMYC Analysis

The GMYC analysis uses the information contained in a tree to delimit species and determine the diversification model. To analyze the influence of the priors chosen, we constructed 3 different trees from the same DNA sequence alignment, changing the model used to express the branching pattern of the tree (the Yule or a coalescent model) and the rate of molecular evolution, considering a constant or a relaxed clock, as recommended by Michonneau (unpublished). The topology of the trees would be almost identical in all cases; however, the branch lengths would vary.

The analysis was conducted using the function gmyc available in the splits package implemented in R (Ezard et al., 2014), with the single threshold option as recommended by Fujisawa and Barraclough (2013). The method compares the null (no threshold) and alternative hypothesis (one threshold) and infers the number of genetic entities. Branching events between species are modeled with a Yule model (Barraclough and Nee, 2001), while branching events within species are adjusted to a neutral coalescent process (Hudson, 1991).

Results are evaluated by a likelihood ratio test (LRT) between the null hypothesis, which considers that all strains analyzed constitute a single species, and the alternative hypothesis (GMYC model) that assumes the existence of a species-delimiting threshold. The LRT significance was calculated using a chi-square test with 2 degrees of freedom.

The gmyc function gives a likelihood score for the model that considers all sequences belonging to the same species, and a likelihood score considering the sequences split in different species. The output also lists how many clusters and entities are associated with the highest likelihood score with the corresponding confidence intervals (CI) and the estimated threshold time when there was a transition between the speciation- and coalescent-level events. The R package also contains functions that plot (1) the number of lineages-through-time (LTT), with the inferred position of the threshold (red vertical line); (2) the likelihood profile through time; (3) the tree with the clusters highlighted in red. Additionally, the “support” for the delineated species can be plotted, indicating whether the results are reliable or not.

The use of only one indirect calibration point in the construction of the phylogeny, due to the absence of more reliable calibration data, can be a source of uncertainty. As the GMYC method relies on relative rather than absolute branch lengths, we repeated the analysis with the same alignment but removing the outgroup, obtaining the corresponding tree. Following the suggestion of Fujisawa and Barraclough (2013), we scaled branch lengths in the tree to have a root age of 1.0 before running the analysis.

### Diversification Analyses

A standard lineage-through-time (LTT) plot was constructed using the R package ape (Paradis et al., 2004) to graphically visualize and evaluate the temporal pattern of lineage diversification in *Aeromonas.*

We used the birth–death likelihood (BDL) tests implemented in laser (Rabosky, 2009) to detect the diversification model and the speciation and extinction rates (λ and μ) from the pruned tree obtained by cutting the original tree at the threshold given by the GMYC analysis. To test the null hypothesis of no-rate change versus variable-rate change in diversification, we have applied the ML approach of Rabosky, the test ΔAIC_RC_ (Rabosky, 2009). This statistic is calculated as: ΔAIC_RC_ = AIC_RC_ – AIC_RV_, where AIC_RC_ is the Akaike information criterion (AIC) score for the best fitting rate-constant diversification model, and AIC_RV_ is the AIC for the best fitting variable-rate diversification model. Thus, a positive value for AIC_RV_ indicates that the data are best approximated with a rate-variable model, while a negative AIC_RV_ value suggests a rate-constant model of diversification. We tested eight different models, two of which were rate-constant (pure-birth or Yule and birth-death) and six were rate-variable (DDL, DDX, Yule 2-,3-,4- and 5- rates) (Lorén et al., 2014).

We calculated the gamma (γ) statistic (Pybus and Harvey, 2000; Fordyce, 2010) and its significance by simulating 5,000 phylogenies, as described previously (Lorén et al., 2014). This statistic compares the relative node positions in a phylogeny with those expected under a constant diversification rate model, in which the statistic follows a standard normal distribution. Positive γ values evidence that nodes are closer to the tips than expected under the constant rate model. When γ is negative, the internal nodes are closer to the root than expected under a constant model, indicating a decrease in diversification through time. In addition, we compared the observed empirical gamma value with a distribution of the gamma statistics obtained by simulation.

## Results

### Sequence Analysis

The analysis involved 147 *Aeromonas* strains. The number of total positions analyzed was 1,979 bp. All positions containing gaps and missing data were eliminated in the construction of the phylogenetic tree.

**FIGURE 1:**
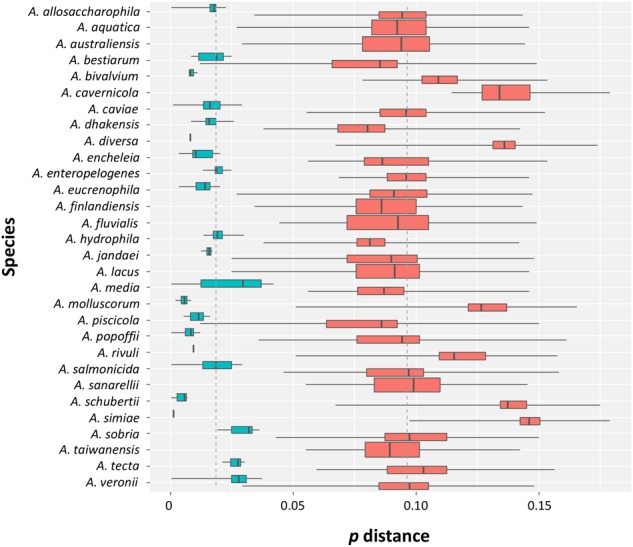
Intra- and inter-specific distances in *Aeromonas* species. Box plots showing the intra- (turquoise) and inter-specific (salmon) *p* distances obtained from the data set. The ends of the boxes correspond to the first and third quartiles. The ends of the horizontal lines indicate the highest and lowest values. The black vertical line dividing the boxes represents the median of the data.

The intra- and inter-specific distances determined from the sequences (Supplementary Figure S1 and Supplementary Table S3), with the exception of the conflicting *A. veronii* group and the *A. bestiarum*/*A. piscicola* cluster, allowed a good discrimination of the species with no overlap among the distances (**Figure 1**).

**FIGURE 2:**
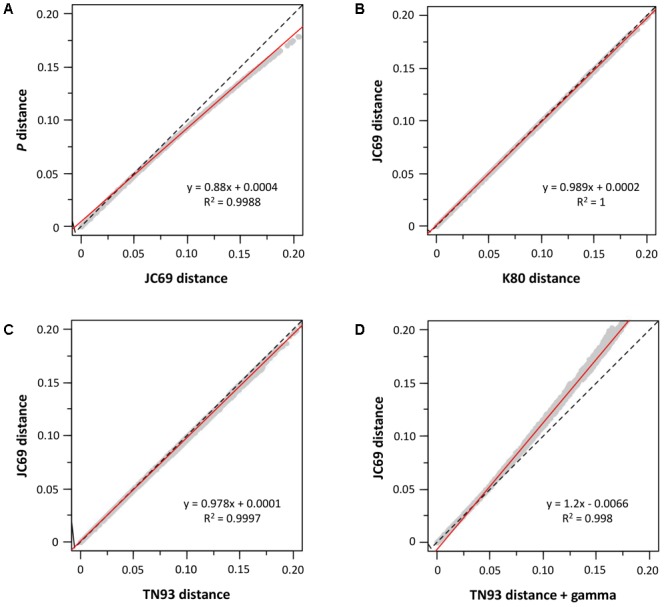
Sequence analysis. Graphs **(A–D)** show the influence of different substitution models. The dashed line represents the equation y = x, and the red continuous line is the regression line whose equation appears in the corresponding graph.

We also determined the sequence evolution of the concatenated sequences of *mdh* and *recA.* As can be seen in **Figure 2**, we analyzed our sequences for the influence of saturation (**Figure 2A**), base frequencies (**Figure 2B**), transitions and transversions (**Figure 2C**), and the gamma correction for multiple substitutions (**Figure 2D**). The sequences showed no influence of saturation, base frequency bias or the transition-transversion ratio; only the heterogeneity in the inter-site substitution rate could have some effect (**Figure 2D**).

**FIGURE 3:**
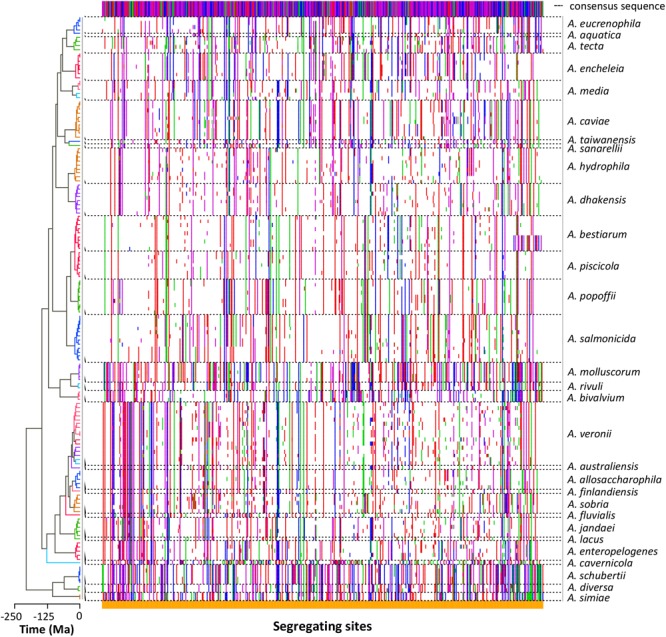
Sequence polymorphism. The dots plot shows the segregating sites determined in each sequence arranged in the same order as in the tree **(left)**. Polymorphic differences between the sequences and the consensus sequence **(top)** are marked in colors depending on the base (A green, G blue, C purple, T red). Dashed lines delimit the sequences belonging to the same *Aeromonas* species.

The nucleotide substitution analysis (**Figure 3**) showed a clear diversity among the sequences of the strains belonging to the same species. Nevertheless, the different species exhibited segregating site patterns that allowed them to be separated.

### Phylogenetic Reconstruction

**FIGURE 4:**
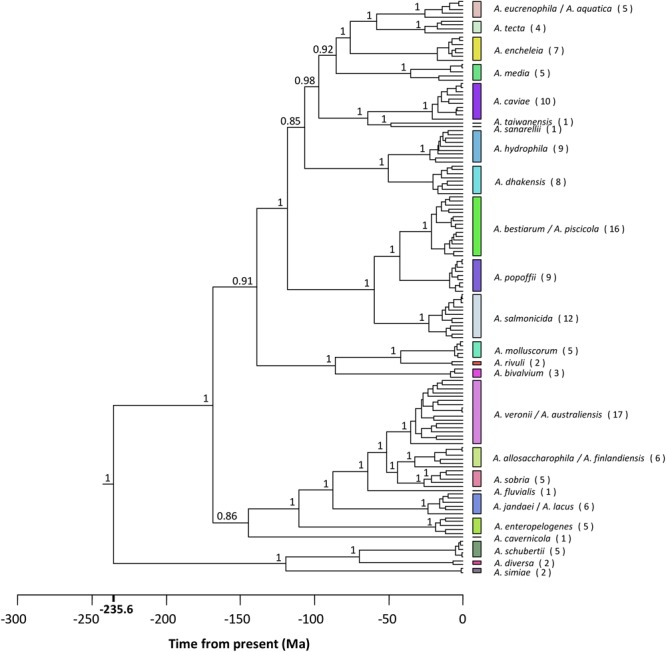
*Aeromonas* phylogeny. Bayesian chronogram with the posterior probability values for the main clusters. Colored vertical bars on the right correspond to the clusters obtained by the NbClust analysis (Supplementary Table S3). The number of strains belonging to the same species is given in brackets. Scale bar at the bottom indicates the divergence time in millions of years (Ma, Mega annum).

The best-fit models of sequence evolution were implemented according to the Akaike Information Criterion (AIC) scores for the substitution models evaluated, using jModeltest. The general time reversible (GTR) model was selected as the best model of evolution for the concatenated sequences using a discrete gamma distribution and a fraction of invariable sites (GTR + G + I). **Figure 4** shows the *Aeromonas* Bayesian phylogeny, all the strains belonging to the same species clustering together in the same group. The posterior values obtained for each node were close to 1 for the majority of the main clades. The figure also shows the clusters obtained with the *p* distance analysis (Supplementary Table S2), which fully matched those established from the phylogeny.

**FIGURE 5:**
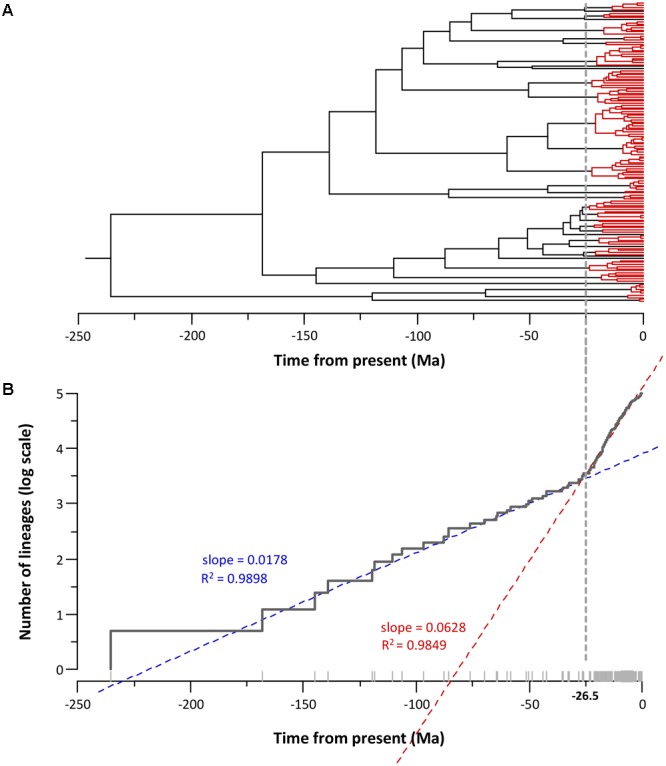
*Aeromonas* diversification. **(A)** *Aeromonas* chronogram showing the temporal node distribution. Nodes posterior to –25 Ma (breakpoint) are marked in red. **(B)** LTT plot corresponding to the chronogram showing the breakpoint and the linear adjustment of the two segments (before and after the breakpoint).

**Figure 5** depicts the phylogeny obtained with the corresponding lineage-through-time plot (LTT plot). The LTT plot (**Figure 5B**) clearly shows two different linear relationships with a sudden change in the slopes at approximately -25 Ma. This breakpoint roughly matches the region of the tree between -25 Ma and the present (**Figure 5A**), which accumulates the majority of the nodes (75%). We applied a linear regression model to the LTT plot points between the crown age (-235.6 Ma) and the breakpoint (-25 Ma) (segment I), and those obtained from the breakpoint to the present (segment II). The fit of the two segments to a linear model was very good, with R squared values of 0.9898 and 0.9849 for segments I and II, respectively. The two regression lines intersected at -26.5 Ma, and the slopes for segment I and II were 0.0178 and 0.0628 (**Figure 5B**). Using the Chow test (Chow, 1960), we verified that the two linear regression slopes differed significantly (*F* test = 1715.8, *P* value = 1.12e^-100^).

### Generalized Mixed Yule Coalescent

**FIGURE 6:**
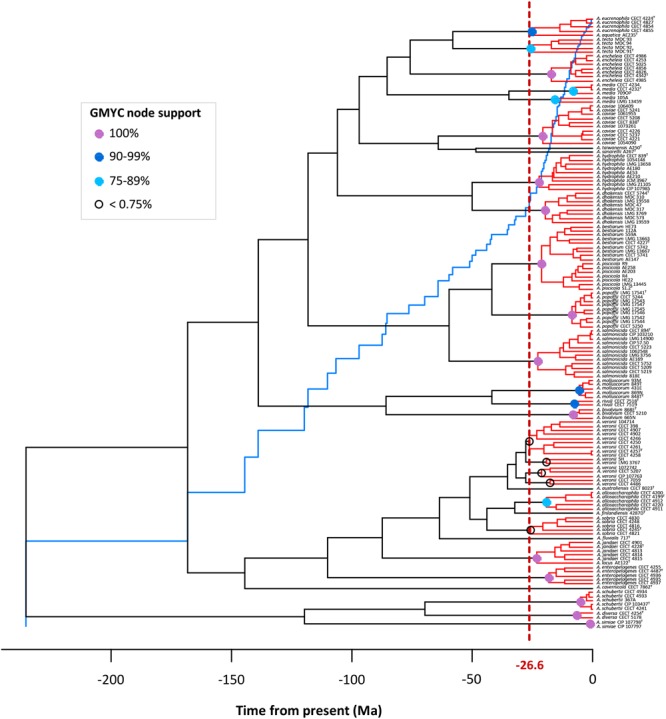
GMYC analysis. The LTT plot (blue line) showing a clear abrupt change from the threshold (vertical dashed red line). Node support of the main GMYC clusters (red) is illustrated by colored circles (see legend in box).

The existence of distinct *Aeromonas* lineages was confirmed by the branch length analysis. The resulting LTT plot showed a steep upturn in branching rates toward the present, marking the transition from the between- to within-species rate of lineage branching (**Figure 6**). The GMYC model fitted a transition in branching rate occurring at -26.6 Ma. The support in the majority of the GMYC clusters was high, confirming the reliability of the results (**Figure 6**). The number of ML entities determined was 31 with a confident interval (CI) of 24–36 (**Table 1**). The λ values for the diversification and coalescent processes were 0.0092 and 0.2779, respectively. The scaling parameter p for the diversification process was close to 1 (1.154), which indicates a constant speciation rate model with no extinction (Yule model), while the value for the coalescent process was clearly below 1 (6.186e^-08^) indicating a deficit of recent coalescent events (Fujisawa and Barraclough, 2013). No substantial differences were detected between Bayesian models using a coalescent or Yule prior when constructing the tree (**Table 1**).

**Table 1:** GMYC analysis.

	GMYC	
	Yule	Coalescent
	Constant clock	Constant clock
Number of ML entities (CI)^a^	31 (24 – 36)	31 (25 – 38)
Threshold time	–26.60	–24.60
pdiv value^b^	1.1548	1.2170
pcoal value^c^	6.1860e-08	2.0497e-09
λ_div_^b^	0.0092	0.0080
λ_coal_^c^	0.2779	0.3026

*^*a*^ML, maximum likelihood; CI, confidence interval. ^*b*^div, diversification process. ^*c*^coal, coalescent process.*

The calibration point used to date the phylogeny also had no influence. Identical results were obtained when the dated tree was compared with the one obtained by repeating the analysis with the same alignment but removing the outgroup.

### Diversification Analysis

As suggested by Rabosky (2006), we calculated the significance of ΔAIC_RC_ for the set of analyzed models by using the Yule model to simulate 5,000 phylogenies of the same size and diversification rate as those obtained from our data, and determined the *P* value from the resulting distributions. As can be seen in **Table 2**, we cannot reject the null hypothesis of a Yule model to a level of significance of α = 0.05, which means that the diversification in *Aeromonas* is constant.

**Table 2:** Diversification models.

Models	AIC^a^								
	Pure birth	Birth-death		DDX	DDL	Yule-2-rates	Yule-3-rates	Yule-4-rates	Yule-5-rates
	150.55	151.83		152.21	152.55	152.80	152.30	153.02	155.56

**ΔAIC_RC_ test^b^**	**Best constant model**		**Best variable model**		**ΔAIC_RC_**	***P* value^c^**	**Best model**		**λ (ML)**
	
	Pure birth		DDX		–1.6569	0.753	Pure birth (Yule)		0.017

*^*a*^ AIC, Akaike Information Criterium. ^*b*^ΔAIC_*RC*_ test (Rabosky, 2006). ^*c*^*P* value obtained by simulation of 5,000 phylogenies.*

**FIGURE 7:**
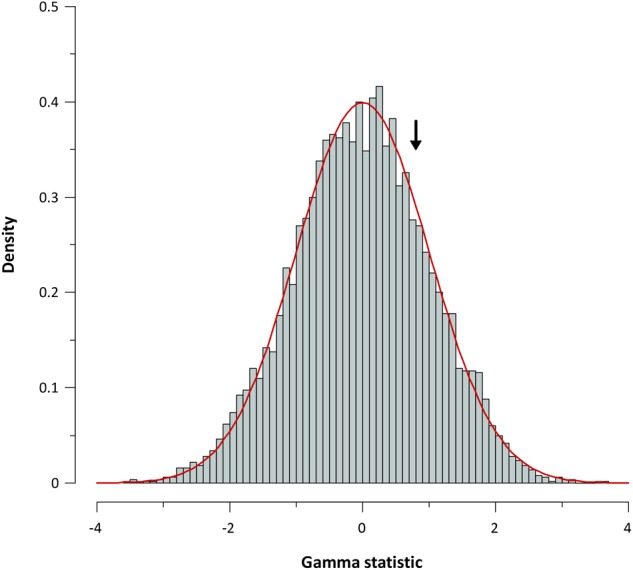
Gamma statistic distribution. Gamma statistic distribution obtained by simulating 5,000 phylogenies under the Yule model. The arrow indicates the empirical gamma (γ) value obtained.

For further corroboration, we determined the gamma statistic of Pybus and Harvey, a powerful tool principally used for comparing models of decreasing speciation rate through time and a constant rate of diversification. The estimated γ value from the chronogram (pruned tree) was 0.798 with a *P* value of 0.425, indicating that we cannot reject the null hypothesis of constant diversification for our phylogeny. Data obtained from the simulation also corroborate a constant diversification process in *Aeromonas* (**Figure 7**).

## Discussion

The choice of appropriate genes is crucial for the reconstruction of reliable molecular phylogenies. They should be housekeeping genes, evolving at a constant rate (good molecular clocks), and have unsaturated synonymous and non-synonymous substitutions. Unlike the majority of phylogenetic studies, in our analysis we checked our sequences to ensure that the genes used in this work fulfilled the above conditions and allowed a good species separation.

The phylogeny constructed from the concatenated sequences corroborates the monophyletic origin of this group of bacteria. In the chronogram obtained, the majority of the nodes were strongly supported, with posterior values close to 1. In addition, the main clade distribution was in agreement with previously published phylogenies (Roger et al., 2012; Colston et al., 2014; Lorén et al., 2014; Sanglas et al., 2017). We obtained a perfect clustering of the strains belonging to the same species, including those considered synonymous.

The analyses based on the LTT plot detected an increase in the diversification rates from a certain point in time. As **Figure 5** illustrates, a high percentage of nodes accumulate close to the present due to the greater similarity of the sequences belonging to the same species.

The LTT plot constructed from the phylogenetic tree clearly shows two different linear relationships with a sudden change in the slopes at -26.5 Ma. The fit of the two segments to a linear model was very good, with high *R* squared values. The Chow test verified that the two linear regression slopes differed significantly (*P* value = 1.12e^-100^), which indicates a change in the rate of lineage branching.

In this work we carried out a quantitative analysis of sequence data to delimit putative *Aeromonas* species by detecting shifts in the rate of lineage branching. The branch-length analysis is based on a probabilistic model that distinguishes between species diversification and coalescent processes. This approach compensates for undefined species limits by including confidence intervals when allocating species-defining nodes and does not rely on population limits; thus, species represented by a single strain can be included (Pons et al., 2006).

Some studies recommend the use of BEAST for the tree construction as input for the GMYC analysis (Monaghan et al., 2009; Tang et al., 2014). From a set of given data (sequences) and a substitution model, the Bayesian Inference performs a probability analysis in search of the best set of trees that maximize the posterior probability. From an initial tree, it uses the Markov Chains Monte Carlo to evaluate the posterior probability of the different states proposed. After generating a large number of trees (frequently around 10,000), it uses the subsequent probability to ascertain how many times a node is repeated in each tree and this is done for each node. Subsequently, the Bayes theorem is used to build a single phylogenetic tree (maximum clade credibility, MCC). The same method provides an estimate of the reliability of the results obtained through the posterior probability values of each node, without the need to evaluate it *a posteriori*.

Although Monaghan et al. (2009) recommended the use of the coalescent tree prior instead of the Yule prior for constructing the tree for the GMYC analysis, in our work, in agreement with Talavera et al. (2013), we obtained identical results with the different BEAST options.

Barraclough et al. (2009) used the GMYC method to delimit bacterial species based on 16S rRNA sequences, concluding that the 16S rRNA is a rather conservative molecule for surveying bacterial diversity and cannot be used to distinguish putative species. They suggest that studies using sequences of multiple genes or whole genomes of individuals would be more useful. Accordingly, we applied the GMYC method to delimit species in the genus *Aeromonas*, and the results corroborate the species previously described with other methods (phenotypic analysis, MLSA, genome sequence analysis) for this bacterial genus. The resulting tree exhibited the branching rate pattern of the species-to-population transition. Only the controversial *A. veronii* and *A. media* species complexes, the *A. piscicola*/*A. bestiarum* group, and two of the most recently proposed species were not entirely resolved.

**FIGURE 8:**
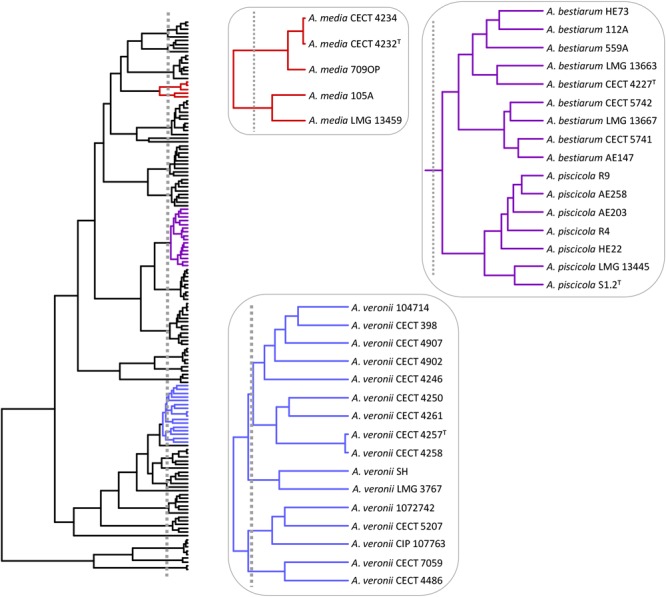
*Aeromonas* species delimitation. Phylogenetic tree obtained with the GMYC method. In boxes are the species clusters that present taxonomic uncertainties when using the estimated breakpoint to delimit species.

The *A. veronii* group includes two biovars, *A. veronii* Veronii and *A. veronii* Sobria, as well as two other *Aeromonas* species considered as synonymous, *A. culicicola* (Huys et al., 2005) and *A. ichthiosmia* (Huys et al., 2001). When we analyzed the *A. veronii* species complex, four groups of strains were revealed: a main cluster consisting of 9 strains, including the two biovars, *A. veronii* Veronii and *A. veronii* Sobria; a second group with two strains, including *A. ichthiosmia*; a third comprising three strains, including *A. culicicola*; and the fourth with two strains (**Figure 8**).

Considering the GMYC results, it would be interesting to review the inclusion of *A. culicicola* and *A. ichthiosmia* in the already controversial *A. veronii* group (Huys et al., 2001; Esteve et al., 2003). In addition, Colston et al. (2014) determined the ANI and *is*DDH values from the genomes of different *Aeromonas* species. When the authors compared the type strain of *A. culicicola* and *A. ichthiosmia* with all the strains defined as *A. veronii*, the *is*DDH values obtained were below the threshold of 70% (69.1–69.6% for *A. culicicola* and 67.4–68.2% for *A. ichthiosmia*) and ANI values close to 96%, suggesting these species are different from *A. veronii*.

*Aeromonas media* described by Allen et al. (1983) traditionally comprised two hybridization groups: *A. media* HG5A and HG5B (Hänninen and Siitonen, 1995), with the strains LMG 13459 (CDC 0862-83) and CECT 4232 (ATCC 33907) representing both groups (Popoff et al., 1981). This was later corroborated by Küpfer et al. (2006) and more recently by Talagrand-Reboul et al. (2017). Our results reveal the existence of two groups of strains outside the GMYC threshold that determine the species in *Aeromonas* (**Figure 8**), which is in accordance with Talagrand-Reboul et al. (2017), who analyzed the relationships of 40 *A. media* strains with the recently proposed species of *A. rivipollensis*. These authors also identified two groups of strains inside the *A. media* cluster with ANIb (ANI calculator and EzGenome) and *is*DDH values between groups of ≤94.6% and ≤61.2%, respectively. These values, which are below the considered threshold for isolates of the same species, support the existence of two different species within this group.

Another controversial GMYC clade grouped together *A. bestiarum*/*A. piscicola* (**Figure 8**). Colston et al. (2014) determined 0.04 substitutions per site for this pair of strains, which was clearly lower than those determined for other *Aeromonas* species, such as *A. eucrenophila*/*A. tecta* (1.0) or *A. schubertii*/*A. diversa* (0.9). Furthermore, the ANI or *is*DDH values for *A. bestiarum*/*A. piscicola* were close to the threshold to be considered as members of the same species.

Recently, three new *Aeromonas* species have been formally accepted: *A. finlandensis, A. lacus* and *A. aquatica* (Beaz-Hidalgo et al., 2015). Nevertheless, our analysis showed that *A. lacus* and *A. aquatica* should probably not be considered new species (**Figure 6**). In the case of *A. lacus*, the results obtained are in agreement with the ANI values calculated from the genomes in the original description of these species. When *A. jandaei* was compared with *A. lacus*, the ANI value obtained was 95.38%, similar to the species cutoff of 96%.

The GMYC analysis also reveals that diversification in *Aeromonas* is a constant process and follows a Yule model. This result is independent of the number of sequences per species used, and agrees with a study of macroevolutionary dynamic models in a huge number of eukaryote and prokaryote taxa (Maruvka et al., 2013). Prokaryote populations are less affected by extinction and founder effects than larger less abundant organisms (Lynch and Conery, 2003; Butterfield, 2011). Bacterial species may be described as metapopulations extending over time and evolving separately from other species (Cohan, 2002a,b; Achtman and Wagner, 2008). Although microbial dispersion can be limited by geographical barriers and the resulting physical isolation may influence microbial evolution, due to their small size bacteria generally have an unrestricted capacity for dispersion (Finlay and Clarke, 1999) via a variety of passive mechanisms and over long distances (Fierer, 2008).

## Conclusion

The GMYC method clearly delineated the species boundaries in *Aeromonas*, even in the controversial groups, such as the *A. veronii* or *A. media* species complexes. Some of these taxonomic uncertainties are due to the use of inappropriate methods for defining species [biochemical tests with miniaturized methods (API) or 16S rRNA sequences]. It would therefore be interesting to revise bacterial species separation using the emerging new analyses based on genome sequences (ANI, *is*DDH). The method presented here, widely used to delimit species in eukaryotes, offers an alternative that is easy to use and fully practicable for all laboratories. In addition, the GMYC method has promising potential for phylogenetic community ecology studies.

## Author Contributions

JGL and MCF conceived and designed the research. JGL and MF performed the computations. JGL, MCF, and MF analyzed the data and discussed the results. All authors contributed to and revised the final manuscript version. They all approved the final version to be published.

## Conflict of Interest Statement

The authors declare that the research was conducted in the absence of any commercial or financial relationships that could be construed as a potential conflict of interest.

## Footnotes

1 http://www.ncbi.nlm.nih.gov/genbank/

2 http://beast.bio.ed.ac.uk/logcombiner

3 http://beast.bio.ed.ac.uk/figtree
