# Supplementary material for: Species Delimitation, Phylogenetic Relationships, and Temporal Divergence Model in the Genus Aeromonas
Source: Front Microbiol. 2018 Apr 20;9:770. doi: 10.3389/fmicb.2018.00770 (PMC5920023; doi:10.3389/fmicb.2018.00770)
Supplement: Supplementary file 1 [file Table_1.PDF]

Table S1. *Aeromonas* strains and gene sequences used in this study.

Species	Strain[a]	Other designation	Source / Geographical origin[b]	GenBank accession no.[c]	
				mdh	*recA*
A. allosaccharophila	CECT 4199[T]	*A. veronii*	Diseased elvers of eel from a fish farm / Spain	HM163292	KM260565
	CECT 4200		Diseased eel from a fish farm / Spain	KM507368	KM260566
	CECT 4220		Faeces / USA	KM507369	KM260567
	CECT 4911		Faeces / Switzerland	KM507370	KM260568
	CECT 4912		Faeces / Switzerland	KM507371	KM260569
A. aquatica	AE235[T]		Lake wàter / Finland	NZ_JRGL01000011.1	NZ_JRGL01000122.1
A. australiensis	CECT8023[T]		Irrigation water / Australia	KM507372	KM260570
A. bestiarum	CECT 4227[T]		Diseased fish	HM163294	KM260547
	112A		Non-drinking water / Spain	JN660159	KM260548
	559A		Drinking water / Spain	JN660162	KM260549
	AE147		Lake water / Finland	JN660167	KM260550
	CECT 5741		Environment / Germany	JN660175	KM260551
	CECT 5742		Water / Switzerland	JN660176	KM260552
	HE73		Water from Iso-Kukka Lake / Finland	JN660179	KM260553
	LMG 13663		Intestine of juvenile silver salmon	JN660183	KM260554
	LMG 13667		Probably water / USA	JN660187	KM260555
A. bivalvium	868E[T]		Cockle / Spain	HM163295	KM260571
	665N		Razor-shell / Spain	KM507373	KM260572
	CECT 5210		Seawater / Spain	KM507413	KM260619
A. cavernicola	CECT 7862[T]	*Aeromonas* sp.	Water of a brook in a cavern / Czech Republic	KM507374	KM260573
A. caviae	CECT 838[T]		Epizootic of young guinea pigs	HM163296	KM260574
	CECT 4221	*A. hydrophila* subsp. *anaerogenes*	Used oil-emulsions	HM163304	KM260575
	106409		Clinical, human / Mallorca, Spain	KM507375	KM260576
	1054090		Clinical, human / Mallorca, Spain	KM507376	KM260577
	1061955		Clinical, human / Mallorca, Spain	KM507377	KM260578
	1073261		Clinical, human / Mallorca, Spain	KM507378	KM260579
	CECT 4226		Oil emulsion	KM507379	KM260580
	CECT 5208		Human abscess / Spain	KM507381	KM260582
	CECT 5237		Human faeces / Spain	KM507382	KM260583
	CECT 5241		Human faeces / Spain	KM507383	KM260584

Species	Strain[a]	Other designation	Source / Geographical origin[b]	GenBank accession no.[c]	
				mdh	***recA***
A. dhakensis	CECT 5744[T]	*A. hydrophila* subsp. *dhakensis*	Child with diarrhoea, faeces / Bangladesh	HM163305	JN660346
	MDC 47	*A. aquariorum*	Aquaria of ornamental fish / Portugal	HM163293	JN660357
	LMG 3769		River water / India	JN660226	JN660347
	LMG 19558		Child with diarrhoea, faeces / Bangladesh	JN660228	JN660349
	LMG 19559		Child with diarrhoea, faeces / Bangladesh	JN660229	JN660350
	MDC 310	*A. aquariorum*	Tropical fish / Portugal	JN660236	JN660358
	MDC 317	*A. aquariorum*	Tropical fish / Portugal	JN660237	JN660359
	MDC 573	*A. aquariorum*	Vega Baja hospital / Spain	JN660238	JN660360
A. diversa	CECT 4254[T]		Human leg wound / USA	HM163298	KM260586
	CECT 5178	*Aeromonas* sp.	Human leg wound / USA	KM507385	KM260587
A. encheleia	CECT 4342[T]		Healthy European eels / Spain	HM163299	KM260588
	CECT 4253	*Aeromonas* sp. HG11	Human ankle suture / New Zealand	HM163300	KM260589
	CECT 4826		Water from an artesian well / United Kingdom	KM507387	KM260591
	CECT 4856		Water from Mohawk River / USA	KM507388	KM260592
	CECT 4985		Hospital environment / Germany	KM507389	KM260593
	CECT 4986		Drinking water well / Finland	KM507390	KM260594
	CECT 5025		Drinking water well / Finland	KM507391	KM260595
A. enteropelogenes	CECT 4487[T]		Human faeces / India	HM163301	KM260596
	CECT 4255	*A. trota*	Human stool / India	HM163325	KM260597
	CECT 4935		Human appendix / USA	KM507392	KM260598
	CECT 4936		Human stool / Thailand	KM507393	KM260599
	CECT 4937		Human stool / Indonesia	KM507394	KM260600
A. eucrenophila	CECT 4224[T]		Fresh water fish	HM163302	KM260556
	CECT 4827		Carp, ascites	KM507395	KM260557
	CECT 4854		Urban well / Germany	KM507397	KM260559
	CECT 4855		Rural well / Germany	KM507398	KM260560
A. finlandiensis	4287D[T]		Lake water / Finland	NZ_JRGK01000350.1	NZ_JRGK01000049.1
A. fluvialis	717[T]		Water from Muga River / Spain	HM163303	KM260601

Species	Strain[a]	Other designation	Source / Geographical origin[b]	GenBank accession no.[c]	
				mdh	*recA*
A. hydrophila	CECT 839[T]	*A. hydrophila* subsp. *hydrophila*	Tin of milk with fishy odour	HM163306	JN660318
	CIP 107985	*A. hydrophila* subsp. *ranae*	Liver of a frog with septicaemia / Thailand	HM163307	JN660345
	1054148		Clinical, human / Mallorca, Spain	JN660200	JN660319
	AE53		Lake water / Finland	JN660203	JN660322
	AE180		Lake water / Finland	JN660206	JN660325
	AE210		Lake water / Finland	JN660207	JN660326
	JCM 3967	*A. hydrophila* subsp. *hydrophila*	NA	JN660217	JN660336
	LMG 13658	*A. hydrophila* subsp. *hydrophila*	Faeces / Switzerland	JN660221	JN660340
	LMG 21105	*A. hydrophila* subsp. *hydrophila*	Waste water lagoon / Morocco	JN660225	JN660344
A. jandaei	CECT 4228[T]		Faeces from patient with diarrhea / USA	HM163309	KM260604
	CECT 4813		Faeces	KM507401	KM260605
	CECT 4814		NA	KM507402	KM260606
	CECT 4815		Faeces	KM507403	KM260607
	CECT 4901		Leg wound / USA	KM507405	KM260609
A. lacus	AE122[T]		Lake water / Finland	NZ_JRGM01000129.1	NZ_JRGM01000175.1
A. media	CECT 4232[T]		Fish farm effluent	HM163310	KM260610
	105A		Non-drinking water / Spain	KM507406	KM260611
	709OP		Large oyster / Spain	KM507407	KM260612
	CECT 4234		Fish farm pond	KM507408	KM260613
	LMG 13459		Infected fish	KM507384	KM260585
A. molluscorum	848T[T]		Wedge-shell / Spain	HM163311	KM260614
	93M		Mussel / Spain	KM507409	KM260615
	431E		Cockle / Spain	KM507410	KM260616
	849T		Wedge-shell / Spain	KM507411	KM260617
	869N		Razor-shell / Spain	KM507412	KM260618
A. piscicola	S1.2[T]		Wild diseased Atlantic salmon / Spain	HM163312	JN660307
	R4		Diseased fish / Spain	JN660191	JN660308
	R9		Diseased fish / Spain	JN660192	JN660309
	AE203		Lake water / Finland	JN660169	JN660285
	AE258		Lake wàter / Finland	JN660171	JN660286
	HE22		Water from Salajärvi Lake / Finland	JN660178	JN660294
	LMG 13445	*A. bestiarum*	Human / Germany	JN660180	JN660296

Species	Strain[a]	Other designation	Source / Geographical origin[b]	GenBank accession no.[c]	
				mdh	*recA*
A. popoffii	LMG 17541[T]		Drinking water production plant / Belgium	HM163313	JN660311
	CECT 5244		Water from Noguera Pallaresa River / Spain	KM507414	KM260620
	CECT 5250		Continental water / Spain	KM507415	KM260621
	LMG 17542		Drinking water production plant / Belgium	JN660194	JN660312
	LMG 17543		Drinking water production plant / Belgium	JN660195	JN660313
	LMG 17544		Drinking water production plant / Belgium	JN660196	JN660314
	LMG 17545		Drinking water production plant / Belgium	JN660197	JN660315
	LMG 17546		Drinking water service reservoir / United Kingdom	JN660198	JN660316
	LMG 17547		Drinking water treatment plant / United Kingdom	JN660199	JN660317
A. rivuli	CECT 7518[T]		Water, karst region / Germany	JN215542	KM260622
	CECT 7519		Water, karst region / Germany	KM507416	KM260623
A. salmonicida	CECT 894[T]	*A. salmonicida* subsp. *salmonicida*	Atlantic salmon from Cletter River / United Kingdom	HM163317	JN660361
	CECT 5752	*A. salmonicida* subsp. *pectinolytica*	Water from a cistern / Spain	HM163316	JN660399
	CIP 103210	*A. salmonicida* subsp. *masoucida*	Sakuramasou, heart blood	HM163315	JN660398
	LMG 14900	*A. salmonicida* subsp. *achromogenes*	Brown trout from Dee River / United Kingdom	HM163314	JN660397
	818E		Cockle / Spain	JN660249	JN660372
	1062548		Clinical, human / Mallorca, Spain	JN660252	JN660375
	AE169		Seawater from Baltic Sea / Finland	JN660253	JN660376
	CECT 5209	*A. salmonicida* subsp. *salmonicida*	Seawater / Spain	JN660258	JN660381
	CECT 5219		Cake / Spain	JN660262	JN660385
	CECT 5223		Mussel / Spain	JN660265	JN660388
	CIP 57.50		Quality control strain	JN660271	JN660394
	LMG 3756		Human, clinical	JN660272	JN660395
A. sanarellii	A2-67[T]		Clinical, wound / Taiwan	HM163319	KM260624
A. schubertii	CIP 103437[T]		Human forehead abscess / USA	HM163320	KM260625
	367A		Aquaculture / South Africa	KM507417	KM260626
	CECT 4241		Forehead abscess	KM507418	KM260627
	CECT 4933		Leg wound of a 47 years old man	KM507419	KM260628
	CECT 4934		Leg wound of a 36 years old woman	KM507420	KM260629

Species	Strain[a]	Other designation	Source / Geographical origin[b]	GenBank accession no.[c]	
				mdh	*recA*
A. simiae	CIP 107798[T]		Monkey faeces / France	HM163321	KM260630
	CIP 107797		Monkey faeces / France	KM507421	KM260631
A. sobria	CECT 4245[T]		Carp / France	HM163322	KM260632
	CECT 4248		Fish	KM507422	KM260633
	CECT 4816	*Aeromonas* sp.	Burbot kidney	KM507433	KM260646
	CECT 4821		Pike	KM507424	KM260635
	CECT 4830		Fish	KM507425	KM260636
A. taiwanensis	A2-50[T]		Clinical, wound / Taiwan	HM163323	KM260637
A. tecta	MDC 91[T]		Faecal sample of a 5 years old child / Switzerland	HM163324	KM260561
	MDC 92		Surface swab of a rainbow trout	KM507426	KM260562
	MDC 93		Tap water	KM507427	KM260563
	MDC 94		Faecal sample of an asymptomatic adult	KM507428	KM260564
A. veronii	CECT 4257[T]	*A. veronii* bv. Veronii	Sputum of drowning victim / USA	HM163327	KM260638
	CECT 4246	*A. veronii* bv. Sobria	Infected frog suffering from 'red leg' disease	HM163326	KM260639
	CIP 107763	*A. culicicola*	Mid gut of *Culex quinquifasciatus* / India	HM163297	KM260640
	CECT 4486	*A. ichthiosmia*	Surface water	HM163308	KM260641
	104714		Clinical, human / Mallorca, Spain	KM507429	KM260642
	1072742		Clinical, human / Mallorca, Spain	KM507430	KM260643
	CECT 398		Human faeces of a child with diarrhoea / USA	KM507399	KM260602
	CECT 4250		Fish	KM507423	KM260634
	CECT 4258		Diarrheic stool / USA	KM507431	KM260644
	CECT 4261		Maxillary sinus / USA	KM507432	KM260645
	CECT 4902		Environment / Germany	KM507434	KM260647
	CECT 4907		Faeces / Switzerland	KM507435	KM260648
	CECT 5207		Human blood / Spain	KM507380	KM260581
	CECT 7059		Drinking water supply / Spain	KM507436	KM260649
	LMG 3767		Human / India	KM507400	KM260603
	SH	*A. culicicola*	Mid gut of *Culex quinquifasciatus* / India	KM507438	KM260651

[a] T, type strain; CECT, Spanish Type Culture Collection; CIP, the Collection of Institut Pasteur; JCM, Japan Collection of Microorganisms; LMG, Laboratorium voor Microbiologie, Universiteit Gent, Gent, Belgium

[b] NA, not available

[c] Data for three species (*A. aquatica, A. finlandiensis, A lacus*) were downloaded from public database (GenBank at NCBI).
