# Supplementary material for: Species Delimitation, Phylogenetic Relationships, and Temporal Divergence Model in the Genus Aeromonas
Source: Front Microbiol. 2018 Apr 20;9:770. doi: 10.3389/fmicb.2018.00770 (PMC5920023; doi:10.3389/fmicb.2018.00770)
Supplement: Supplementary file 4 [file Image_1.PDF]

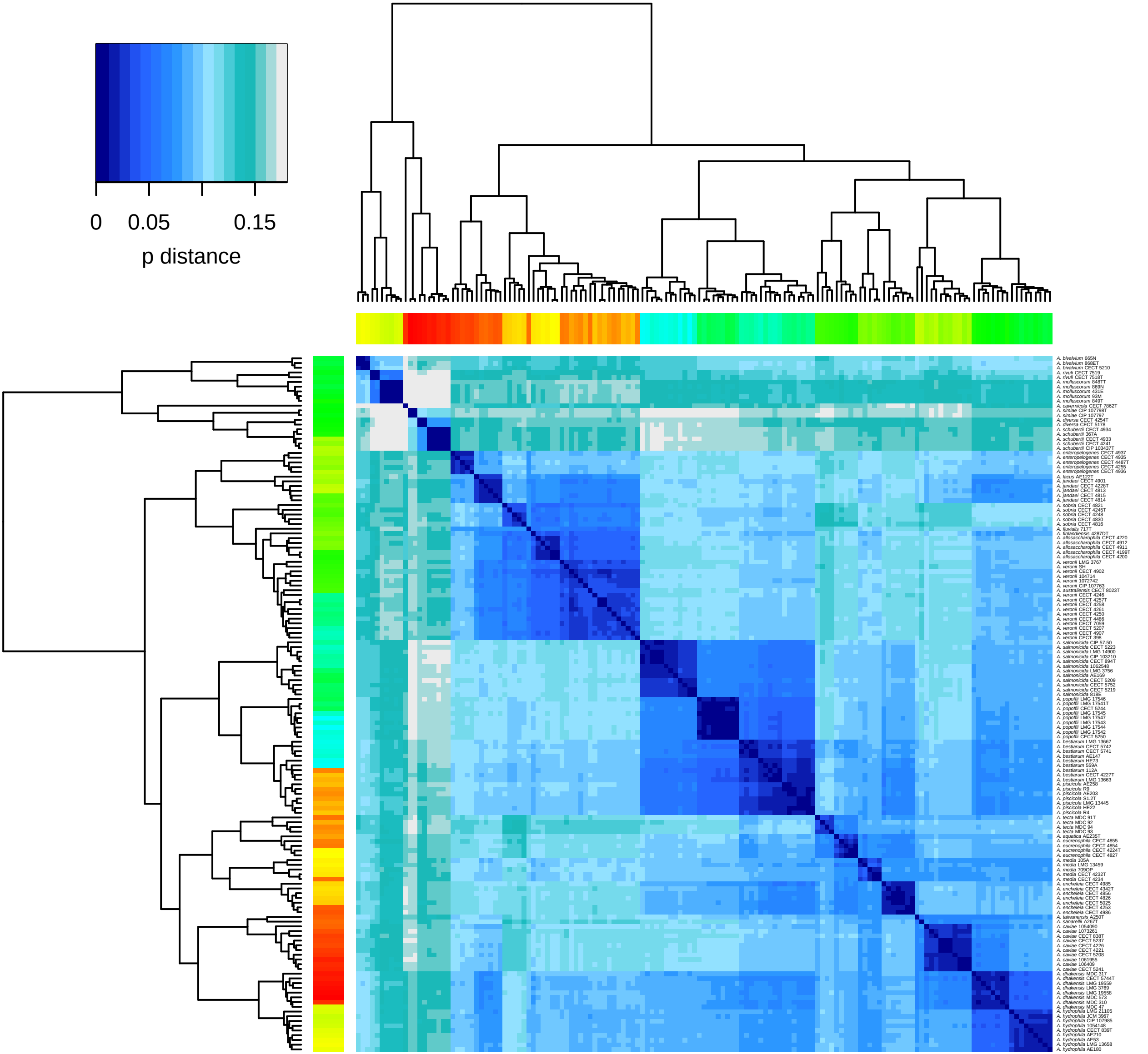

Figure S1. Cluster analysis distance matrix.
